# Influence of Rivet Diameter and Pitch on the Fatigue Performance of Riveted Lap Joints Based on Stress Distribution Analysis

**DOI:** 10.3390/ma13163625

**Published:** 2020-08-16

**Authors:** Jintong Liu, Anan Zhao, Zhenzheng Ke, Zhendong Zhu, Yunbo Bi

**Affiliations:** 1 State Key Laboratory of Fluid Power and Mechatronic System, College of Mechanical Engineering, Zhejiang University, Hangzhou 310027, China; liujintong@zju.edu.cn; 2 Key Laboratory of Advanced Manufacturing Technology of Zhejiang Province, College of Mechanical Engineering, Zhejiang University, Hangzhou 310027, China; kzzcaen@zju.edu.cn (Z.K.); 21725021@zju.edu.cn (Z.Z.); 3 Aviation Industry Corporation of China Xi’an Aircraft Industry (Group) Limited Company, Xi’an 710089, China; zhaoaa@avic.com

**Keywords:** riveted lap joint, stress distribution, fatigue performance, finite element modeling

## Abstract

Interference-fit riveting is one of the most widely used mechanical joining ways in aircraft assembly. The fatigue performance of riveted joints has a significant impact on the service life and reliability of aircraft. In this paper, the fatigue performance of the riveted lap joints with various rivet diameters and pitches are studied based on stress distribution analysis under tensile load. First, a theoretical model of the riveted lap joint under tensile load is developed by using the spring-mass model. The rivet-load stress, bypass stress, and interference stress around the riveted hole are analyzed. Then, the finite element (FE) model of riveted lap joints are established. The influence of rivet diameter and pitch on stress distribution around the riveted hole are discussed. Finally, the fatigue tests are conducted with riveted lap joint specimens to verify the theoretical model and FE results, and a good agreement is observed. Based on the simulation and experimental results, a good combination of structural parameters of the riveted lap joint is found which can optimize the stress distribution around the riveted hole and improve the fatigue life of the riveted lap joint.

## 1. Introduction

With the rapid development of air traffic, fuel consumption and CO_2_ emissions are becoming a growing concern in the field of environmental protection. To make the air traffic sustainable, the lightweight components made of aluminum alloy, titanium alloy, or fiber-reinforced composites have been widely used in aircraft manufacturing [1,2]. These aircraft structures are often joined by riveting, welding, bolting, or hybrid joining technologies [3,4,5], which is a unique challenge in terms of joint reliability, strength, and cost-efficiency. In aircraft manufacturing, riveting is widely used because of its reliability, easy operation, and low cost [6]. The fatigue life of riveted joints is an essential factor affecting flight safety and aircraft service life. Therefore, it is very necessary to analyze, estimate, and enhance the fatigue performance of riveted joints.

The interference fit provided by rivet expansion introduces the residual stress around the riveted hole. The fatigue performance of interference-fit riveted joints can be effectively improved because of residual stress. Atre et al. [7] established a three-dimensional finite element model of the riveting process to determine the effects of interference and sealant on the residual stress state. The results showed that sealant can increase residual hoop stress in most cases. Rans et al. [8] built a 3D finite element model of installation rivets in the monolithic aluminum sheet. It was found that residual stresses beneath the rivet head were influenced primarily by through-thickness compression of the joined sheets. Zeng et al. [9] studied the residual stress distribution in a plate concerning the influence of initial fit tolerance and the squeeze force. It is concluded that both the increase of squeeze force and initial fit tolerance can lead to an increase in residual stress.

In practice, multi-riveted lap joints are commonly used to meet the strength requirements of aircraft components. However, the load carried by individual rivet is different because of the various distances from the position where the load is applied. Researchers have done a lot of research to analyze and optimize the rivet/bolt load distribution in composite joints. McCarthy et al. [10] proposed a simple analytical approach for modeling the non-linear elastic behavior of three-bolts composite joints. They found that increasing bolt torque on the outer bolts can balance the load distribution while the by-pass stresses also played a significant role in controlling joint failure. Wu et al. [11] proposed an analytical method to calculate stress around interference fit holes on composite pinned plates under tensile load. The effects of ply property and ply stack on stress distribution were observed and discussed. Gray et al. [12] performed an experimental investigation into the effects of joint thickness, laminate taper, and missing fasteners on load distribution of single-lap, multi-bolt, countersunk composite joints. It was concluded that missing fasteners can cause significant losses in load-carrying capacity. Atre et al. [13] built a three-dimensional nonlinear finite element model to investigate the stress state at rivet holes in fuselage lap joints. The critical stress zone at the inner skin lower row rivet hole in finite element model was observed to be consistent with the fatigue cracking location in experiments.

Researchers have devoted much attention to the prediction of fatigue life and the fatigue performance of riveted joints. Yu et al. [14] developed a fatigue life prediction model for multi-rivet structures considering the residual stress and cyclic load. The prediction results were compared with the experimental data and showed a good performance. Skorupa et al. [15] conducted experimental research on the influence of several production factors on the fatigue behavior of riveted lap joint specimens under constant amplitude loading conditions. The results indicated that the fatigue performance of the rivet with the compensator is far superior to the performance of the round head rivet and the universal rivet. Huang et al. [16] developed a fast assessment method to estimate the fatigue reliability of multiple riveted joints. The proposed method was validated by fatigue tests and can be used for risk-based maintenance decisions of riveted lap joint in aircraft structures. Muller [17] performed an extensive investigation on riveted lap joints. It is concluded that the squeeze force-induced local curvature has a large effect on the load transfer and secondary bending and can change the fatigue life to a large extent.

The coupling of the residual stress induced by riveting and the non-uniform load distribution of different rivets has a great influence on the fatigue performance of riveted joints. However, the existing research on improving the fatigue performance of riveted joints is limited to the optimization of riveting process parameters and residual stress distribution around the riveted hole. The distributions of rivet load and bypass load with various rivet sizes and pitches have not been studied, and the effect of the non-uniform load distribution on the fatigue performance of riveted lap joints is still scarcely understood.

To address the above issue, in this paper, the transfer of rivet load and bypass load under tensile load is theoretically analyzed based on the spring-mass model. The finite element models of riveted lap joints with various rivet diameters and pitches are established. The effects of these variations on stress distribution are discussed, and the residual stress around the riveted hole and the non-uniform load distribution are also investigated. The riveting experiments are conducted based on the automatic drilling and riveting system and the fatigue tests are carried out to verify the theoretical model and simulation results. The effects of rivet diameter and pitch on fatigue life of riveted lap joints are discussed.

## 2. Theoretical Basis

### 2.1. Interference-Fit Riveting

**Figure 1:**
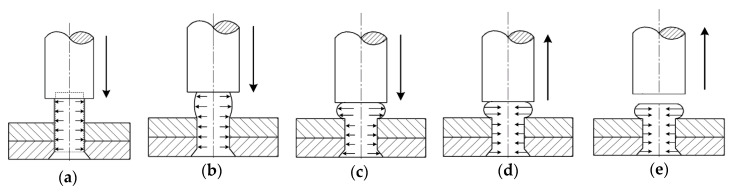
Interference-fit riveting process: (**a**) free upsetting of rivet; (**b**) interference forming with rivet-hole contact; (**c**) driven head forming; (**d**) springing-back; (**e**) punch out of contact with rivet.

As shown in Figure 1, according to the contact and deformation state of the rivet and plates, the process of interference-fit riveting can be divided into five stages:

(1) Free upsetting of the rivet. This stage begins with the punch contacts the upper surface of the rivet, as the movement of punch continues, the rivet shank gradually expands and the rivet material is squeezed into the gap between the rivet shank and hole wall. This stage ends with the rivet shank contacts riveted hole wall.
(2) Interference forming with rivet-hole contact. Under the action of punch movement, the rivet shank continues to be squeezed and the interference between rivet and plates is formed.
(3) Driven head forming. The material flowing in the hole is restricted by the hole wall and the rivet shank is hard to keep expanding. Meanwhile, as the punch continues to press down, the rivet material outside the hole flows in the radial direction to form the driven head.
(4) Springing-back. The punch begins to retreat but remains in contact with the rivet. The elastic deformation of the driven head begins to rebound until the punch is out of contact with the rivet. The squeeze force transmitted by the driven head is reduced, so the plate begins to rebound, too.
(5) Punch out of contact with the rivet. Finally, the residual stress field in the riveting area is formed, the punch is withdrawn and the riveting process is completed.

**Figure 2:**
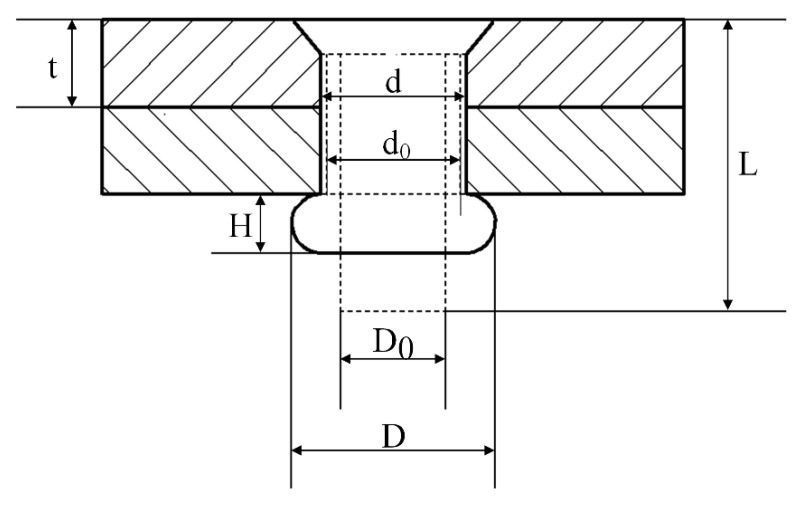
The geometric parameters of the riveted joint: *t*: plate thickness; *L*: rivet length; $d_{0}$: riveted hole diameter before riveting; *d*: riveted hole diameter after riveting; $D_{0}$: rivet diameter; *D*: driven head diameter; *H*: driven head height.

After riveting, the interference and driven head dimensions are regarded as the evaluation indexes of the riveting quality. In practice, the riveting quality is usually verified by measuring the driven head dimensions. Usually, it is considered that the total volume of the rivet material does not change during the riveting process, so the driven head dimensions are related to the interference amount. The definitions of the driven head diameter, height, and interference are shown in Figure 2. 

Assume that the deformation of the rivet expands uniformly along the direction of the rivet shank, according to the law of invariance of volume of the rivet before and after riveting, there is:

(1) $$\frac{\pi }{4}LD_{0}^{2}\approx \frac{\pi }{2}td^{2}+\frac{\pi }{4}HD^{2},$$

where L is the rivet length, $D_{0}$ is the rivet diameter, d is the rivet hole diameter after riveting, t is the plate thickness, H and D are the height and diameter of the driven head, respectively. Then the interference Δ can be obtained as:

(2) $$\Delta =d-d_{0}=\sqrt{\frac{LD_{0}^{2}-HD^{2}}{2t}}-d_{0},$$

### 2.2. Stress Distribution Model of the Multi-Riveted Joint under Tensile Load

In the traditional design of the riveted joint, all rivets and plates are regarded as rigid bodies, each rivet bears the same load, and the static strength of the riveted joint is guaranteed by a safety factor. However, because of the dispersion and sensitivity of the fatigue behavior, the difference of load-bearing capacity of rivets in different positions should be taken seriously. In this section, a stress distribution model of a multi-riveted joint under tensile load is established considering the effects of rivet-load stress, bypass stress, and interference stress. 

**Figure 3:**
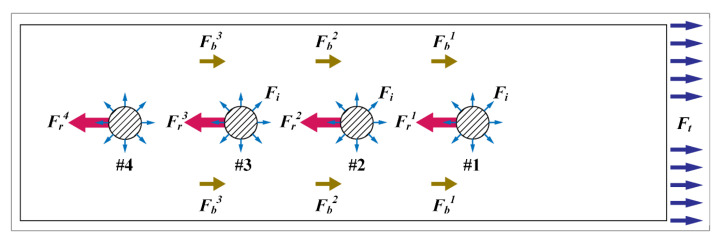
Schematic diagram of the force condition of multi-riveted interference fit joint. $F_{r}^{i}$ represents the rivet load, $F_{b}^{i}$ represents the bypass load, $F_{i}$ represents the interference load, and #i represents the position of the rivet. $F_{t}$ is the tensile load.

The load condition of the riveted lap joint under tensile load can be regarded as fixing one end of the plate and applying the tensile force at the other end. As shown in Figure 3, the stress distribution near the riveted hole will be affected by the rivet-load stress, the bypass stress, and interference stress.

For rivet load, there is:

(3) $$F_{t}=\sum_{i}^{N}F_{r}^{i},$$

where $F_{t}$ is the tensile load, $F_{r}^{i}$ represents the rivet load, and *N* is the number of rivets. 

The bypass load is equal to the sum of the previous rivet load and bypass load. Therefore, the bypass load can be represented as:

(4) $$F_{b}^{i}=F_{b}^{i-1}+F_{r}^{i-1},$$

**Figure 4:**
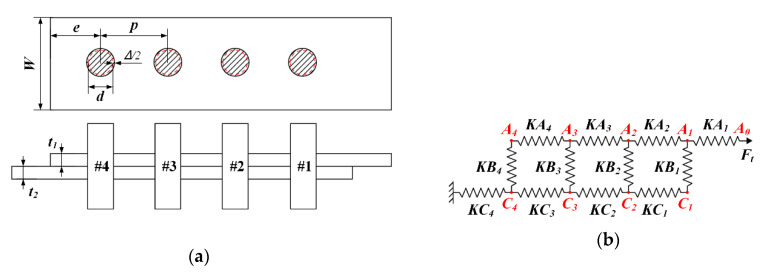
Spring-mass model of the riveted lap joint with four rivets: (**a**) Simplified model, where W represents the plate width, e represents the rivet margin, p represents the rivet pitch, d is the rivet diameter, Δ represents the interference, $t_{1}$ and $t_{2}$ represent the thicknesses of the upper and lower plates, respectively; (**b**) spring-mass model, where $KA_{i}$ represents the spring stiffness of the upper plate, $KB_{i}$ represents the spring stiffness of rivet and $KC_{i}$ represents the spring stiffness of the lower plate.

On the other side, the interference load $F_{i}$ is only related to the amount of interference. Therefore, the rivet load is the key to analyze the stress distribution and load transfer of the riveted joint. The spring-mass model can be used to express the physical process of load transfer when the riveted joint is under tensile load [11,18]. Take the riveted lap joint with four rivets as an example, the load condition of rivets and plates in different positions is analyzed and illustrated in Figure 4.

Since the direction of tensile deformation of plates and shear deformation of rivets are both in the horizontal direction, the plates and rivets can be simplified as springs with elasticity only in the horizontal direction, ignoring the deformation in the axial direction. The springs are connected by a mass point which reflects the actual position where the plates and rivets are squeezed against each other. Define $x^{i}$ as the displacement of mass, according to Hooke’s law, the load transmitted by each spring can be calculated by multiplying the spring stiffness and the displacement difference between the mass points at the ends of the spring.

For interference-fit riveting, the deformation of the riveted hole caused by interference is taken into account, then the load transmitted by each spring can be obtained:

(5) $$\{\begin{array}{c} FA_{i}=KA_{i}\left(x_{A}^{i-1}-x_{A}^{i}-\Delta_{i}/2-\Delta_{i-1}/2\right)\\ FC_{i}=KC_{i}\left(x_{C}^{i}-x_{C}^{i+1}-\Delta_{i}/2-\Delta_{i+1}/2\right)\\ \;FB_{i}=KB_{i}\left(x_{C}^{i}-x_{A}^{i}\right) \end{array},$$

where $FA_{i}$, $FC_{i}$, and $FB_{i}$ represent the load transmitted by each spring, $KA_{i}$, $KC_{i}$, and $KB_{i}$ represent the spring stiffness of upper plate, lower plate, and rivets. $x_{A}^{i}$, $x_{C}^{i}$ represent the displacement of each mass point. $\Delta_{i}$ represents the interference of riveted hole.

The equivalent spring stiffness of plates in Equation (5) is related to geometric parameters and material properties, which can be calculated as follows:

(6) $$\{\begin{array}{c} KA=\frac{E_{p}\times W\times t_{1}}{p-d}\\ KC=\frac{E_{p}\times W\times t_{2}}{p-d} \end{array},$$

where $E_{p}$ represents elastic modulus of the plates, W represents the plate width, p represents the rivet pitch, d represents the rivet diameter, $t_{1}$ and $t_{2}$ represent the thicknesses of the upper and lower plates, respectively.

The equivalent spring stiffness of the rivet in Equation (5) is usually derived from the following empirical formula [11]:

(7) $$\frac{1}{KB}=\frac{2\left(t_{1}+t_{2}\right)}{3G_{b}A_{b}}+\left(\frac{2\left(t_{1}+t_{2}\right)}{t_{1}t_{2}E_{r}}+\frac{t_{1}+t_{2}}{t_{1}t_{2}E_{b}}\right)\left(1+3\beta \right),$$

where $A_{b}$ represents the cross-sectional area of the rivet, $G_{b}$ represents the shear modulus of rivet and $E_{r}$ is the elastic modulus of the rivet. β is the coefficient to describe the secondary bending, which can be seen as zero due to the bending in the axial direction is ignored in this paper.

By solving the load-displacement equation set in Equation (5), the load distribution of riveted lap joint can be obtained. According to Equation (3) and Equation (4), the sum of all rivet loads is equal to the external tensile load F, and the bypass load transmitted backward after the previous rivet is equal to the sum of the external loads of all subsequent rivets. Therefore, there is a great difference between bypass loads at different positions, which will cause the differentiation of the stress distribution in different positions of the riveted lap joint. The stresses around the riveted holes at both ends of the lap joint will be significantly greater than middle positions due to the bypass loads. Meanwhile, Equation (6) and Equation (7) indicate that the cross-sectional area (diameter) of the rivet $A_{b}$ and rivet pitch p will affect the equivalent spring stiffness of the rivets and plates. In other words, the rivet loads and bypass loads can be redistributed by changing the rivet diameter and pitch. Moreover, the stress distribution of the riveted lap joint can be effectively optimized, and the fatigue performance of joints can be enhanced without increasing the manufacturing cost.

The stress distribution model of multi-riveted joint indicates that the distribution of rivet load is not uniform due to the existence of bypass load. Furthermore, the stress distribution of riveted lap joint can be optimized by changing rivet diameter and rivet pitch, thus the fatigue life can be increased. This provides a theoretical basis for the subsequent simulation and experimental research.

## 3. Simulation Study

In this section, several three-dimensional (3D) finite element models of the riveted lap joints with various rivet diameters and pitches are established by Abaqus 6.14. The effects of these variables on the stress distribution of the riveted joints are analyzed, which can provide a basis for the structural optimization and fatigue performance enhancement of the riveted lap joints.

### 3.1. Finite Element Modeling

**Figure 5:**
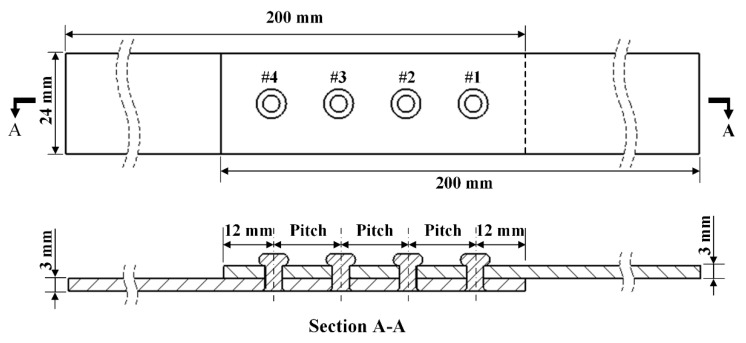
The structure of the riveted lap joint, the rivet diameters are set as 4 mm and 5 mm, the rivet margin is 12 mm, the rivet pitches are 16 mm, 18 mm, or 20 mm.

The structure of the riveted lap joint FE model is shown in Figure 5. Two 2024-T3 aluminum alloy plates and four 2117-T4 aluminum alloy rivets are adopted for simulation. The rivets are numbered from #1 to #4 according to the distance from the applied tensile load. Two sizes of rivets with diameters of 4 mm and 5 mm are selected for simulation and experiment in this paper. According to the manual of aircraft riveting assembly process [19], the rivet pitch should be four times the rivet diameter, so three pitches of 16 mm, 18 mm, and 20 mm are selected for simulation. The rivet margin is set as 12 mm. The size of the plate is 200 mm×24 mm×3 mm.

A total of 10 groups of simulation models are set up with different rivet diameters and pitches, as shown in Table 1. The groups are named “4# rivet diameter_3# rivet diameter_2# rivet diameter_1# rivet diameter_rivet pitch”. The rivet numbers are labeled in Figure 5.

The finite element modeling steps are as follows:

- Geometric model

**Figure 6:**
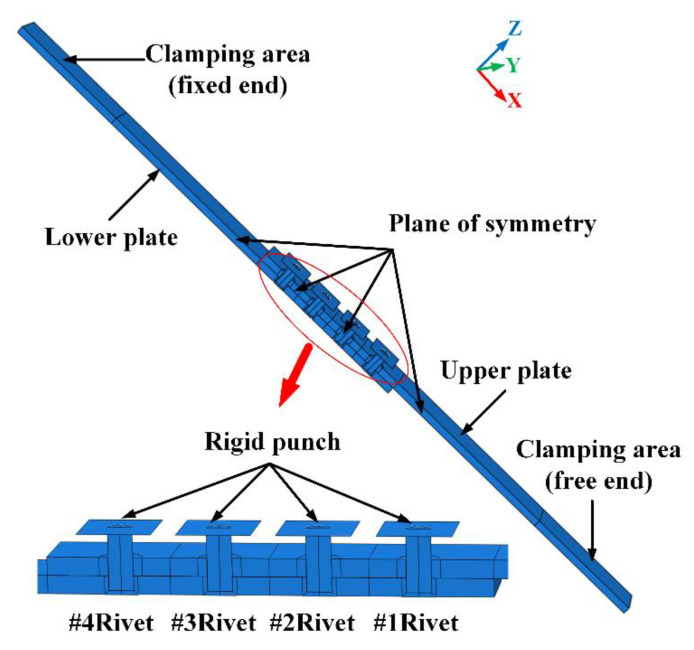
The axisymmetric model of riveted lap joint. There are two kinds or rivet diameters (4 mm and 5 mm) and three kinds of rivet pitches (16 mm, 18 mm, and 20 mm).

As shown in Figure 6, the geometric model of the riveted lap joint mainly consists of four parts: rivets, rigid punch, upper plate, and lower plate. The size of the plate is 200 mm in length, 24 mm in width, and 3 mm in thickness. The rivet type is YSA622¨C100° and the diameters of rivets are 4 mm and 5 mm. There are three rivet pitches: 16 mm, 18 mm, and 20 mm. The punch is simplified as a two-dimensional plane analytical rigid body. As the riveted lap joint is symmetric, a Y-symmetric model is established to reduce the model size and save time.

- Material properties

The materials of the plates are 2024-T3 aluminum alloy and the rivets are made of 2117-T4 aluminum alloy. The material properties are shown in Table 2.

- Mesh selection

**Figure 7:**
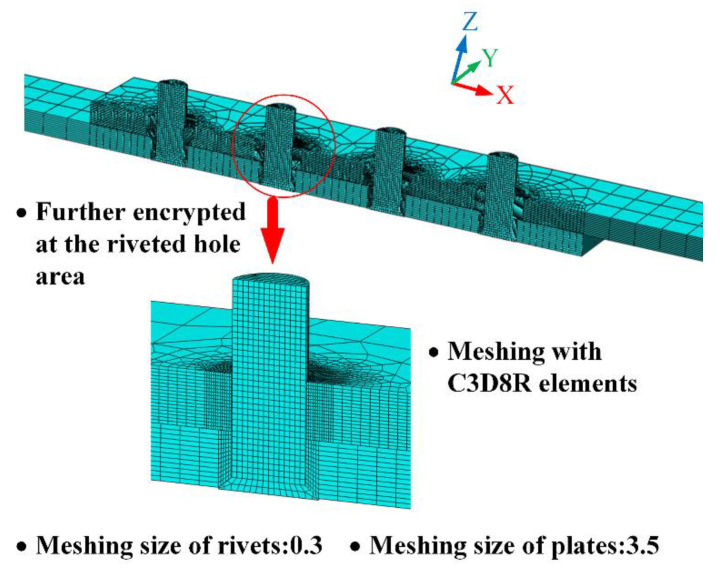
The meshing of the simulation model. The C3D8R elements are used and the riveted hole area is further encrypted.

The meshed model is illustrated in Figure 7. The hexahedral elements of reduced integration eight-node solid continuum elements C3D8R are used for meshing, which can avoid the shear locking problem under large deformation. The meshing size is 0.3 for rivets and 3.5 for plates. Moreover, the meshing of the riveted hole area is further encrypted to obtain a more precise result. Take group 4_4_4_4_16 as an example, the total mesh elements for the riveted lap joint model is 120167.

- Analysis steps

Two analysis steps are set for each rivet: push (stage a to stage c in Figure 1) and release (stage d to stage e in Figure 1). The riveting sequence is #1-#4-#2-#3. After riveting, the tensile load in the X-positive direction is applied to the clamping area on the free end of the upper plate. So, there are a total of 9 steps in the simulation model. Each riveting step time is 10 ms while the tensile step time is 50 ms.

- Load and boundary conditions

The press riveting is controlled by displacement in the finite element model. The displacement is determined by the empirical formula form literature [21]:

(8) $$S=l-\left(t_{1}+t_{2}-H\right)-\frac{D_{0}^{2}l_{2}-d_{0}^{2}\left(t_{1}+t_{2}-H\right)}{D^{2}},$$

where l is the length of the rivet shank, $t_{1}$ and $t_{2}$ are the thickness of the upper and lower plates, respectively, H is the height of rivet head, D is the driven head diameter, $D_{0}$ is the rivet diameter, $d_{0}$ is the hole diameter.

The tension force $F_{b}$ in the last analysis step is determined by the static tensile test. The maximum tangential stress σ on the surface of clamping area can be calculated as:

(9) $$\sigma =\frac{F_{b}}{S},$$

where S is the clamping area.

**Figure 8:**
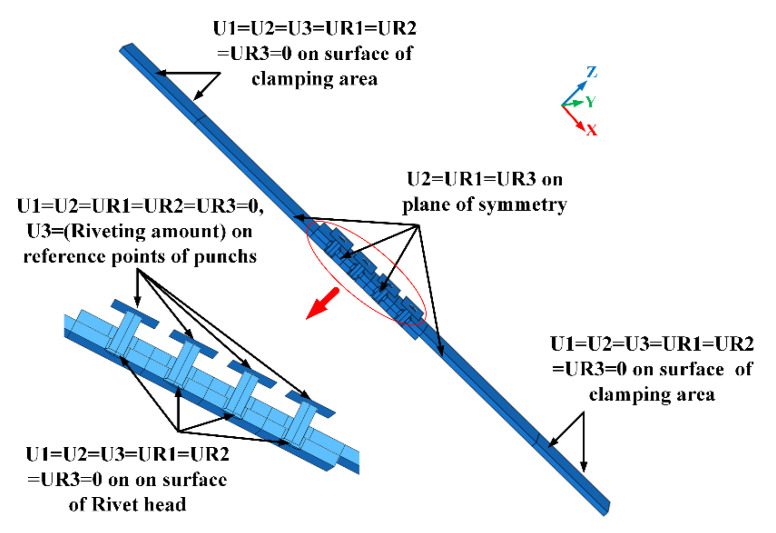
The boundary condition of the finite element (FE) model in the riveting steps.

**Figure 9:**
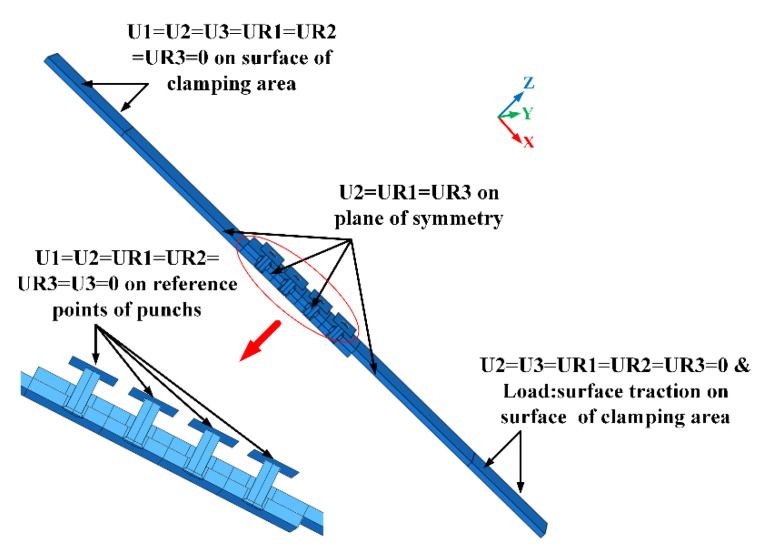
The boundary condition of the FE model in the tensile step.

The boundary conditions in riveting and tensile steps are shown in Figure 8 and Figure 9, respectively. In riveting steps, all degrees of freedom for rivet heads are constrained. The X-direction, Y-direction, and Z-direction are constrained on the clamping areas of the upper and lower plates. The degree of freedom in Z-direction of punches is free so the riveting displacement load can be applied. In the tensile step, all degrees of freedom of punches and the clamping area on the lower plate are constrained. The degree of freedom in X-direction of the clamping area on the upper plate is free for tensile load application. In both riveting steps and tensile step, the translational freedom in Y-direction and rotational freedoms in X and Z-directions are constrained on the symmetry plane.

- Contact and friction settings

The surface interactions in the model mainly include: the contact between punch and rivet, the contact between rivet and hole wall, the contact between the driven head and plate, and the contact between upper and lower plates. The contact pairs were conducted using surface-to-surface with penalty and penetration check in Abaqus. All of these surface interactions are defined as master and slave surface pairs. Coulomb friction with a friction coefficient of 0.2 was adopted.

### 3.2. Simulation Results Analysis

#### 3.2.1. Analysis of Driven Head Dimensions

The driven head dimension is the most direct index to evaluate the riveting quality, and it is widely used in practical engineering. According to the riveting technical standard [19], the driven head dimensions for aluminum alloy rivet should meet the following criteria:

(10) $$\{\begin{array}{c} D=\left(1.5\pm 0.1\right)D_{0}\\ H\geq 0.4D_{0} \end{array},$$

where D is the diameter of the driven head, H is the height of driven head, and $D_{0}$ is the rivet diameter.

**Figure 10:**
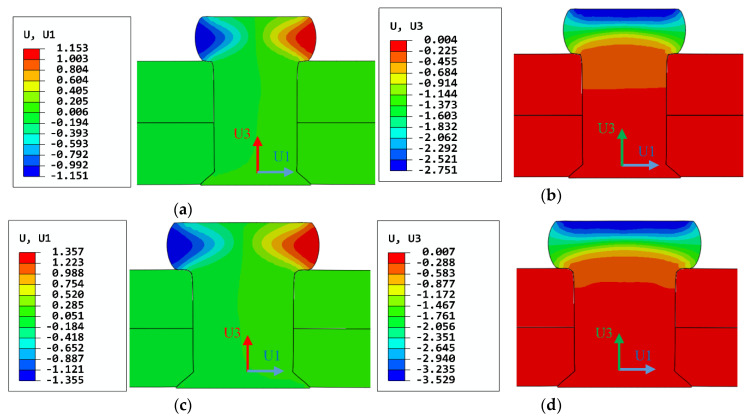
Contours of strains of the rivets after riveting: (**a**) radial strain of the rivet with 4 mm diameter; (**b**) axial strain of the rivet with 4 mm diameter; (**c**) radial strain of the rivet with 5 mm diameter; (**d**) axial strain of the rivet with 5 mm diameter. (unit: mm).

Figure 10 shows the axial and radial deformations of rivets with 4 mm and 5 mm diameters. The simulation results of driven head diameters and heights are shown in Table 3. The results indicate that all the driven head dimensions are meeting the riveting technical requirements. 

#### 3.2.2. Analysis of Interference

**Figure 11:**
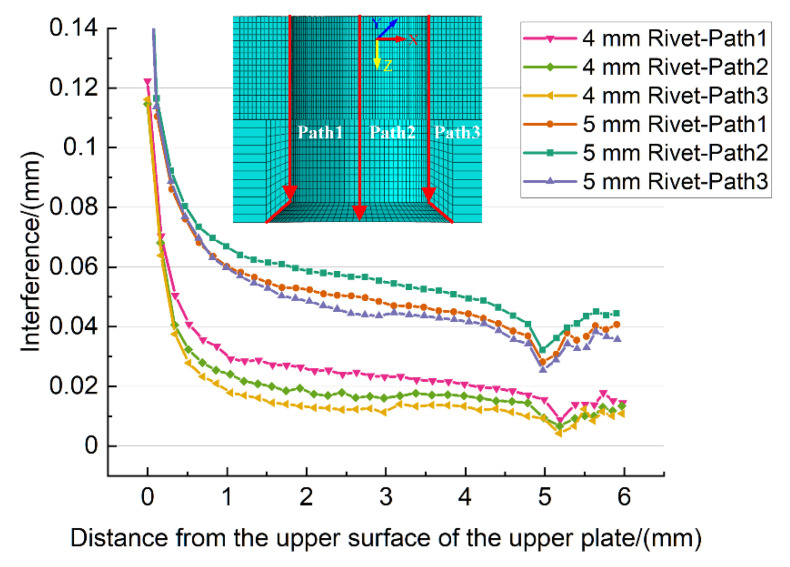
The interferences on the predefined paths along the axial direction of the riveted hole, the X-axis is from the upper surface of the upper plate to the lower surface of the lower plate.

When the rivet begins to deform plastically, the non-uniform interference fit starts to be produced. The interferences of the predefined three paths along the axial direction of the riveted hole are shown in Figure 11. Because of the squeezing effect of the driven head, the interference is the largest near the driven head side, and then rapidly decreases along the paths. For the 5 mm diameter rivet, the turning point of the downward trend appears at about 0.64 mm from the upper surface of the upper plate, where the interference decreases by 62.53%. For 4 mm diameter rivet, the turning point is at 0.52 mm, where the interference decreases by 71.35%. Then the interference is basically stable till the countersunk head side. There is a noticeable fluctuation near the 5 mm in the X-axis, which is at the junction of rivet head and rivet shank. The rivet with 5 mm diameter has a larger countersunk hole depth, so the fluctuation appears earlier than the rivet with a 4 mm diameter. Moreover, the interference caused by 5 mm diameter rivet is significantly greater (more than 2.5 times) than 4 mm diameter rivet. Therefore, the residual stress around the riveted hole induced by 5 mm diameter rivet is better than that with 4 mm diameter rivet.

#### 3.2.3. Analysis of Residual Stress

**Figure 12:**
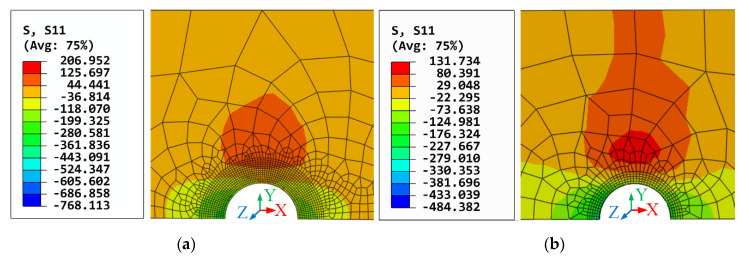

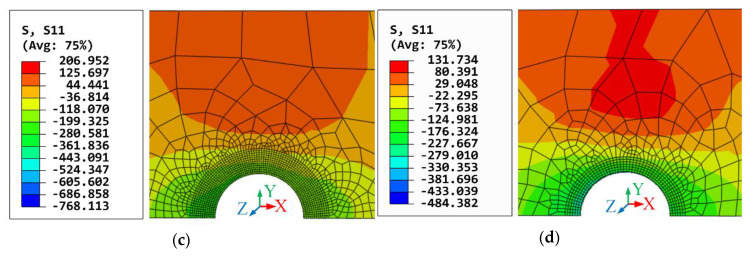
The residual stress distribution of plates: (**a**) The lower surface of the upper plate with 4 mm diameter rivet; (**b**) the upper surface of the lower plate with 4 mm diameter rivet; (**c**) the lower surface of the upper plate with 5 mm diameter rivet; (**d**) the upper surface of the lower plate with 5 mm diameter rivet.

The residual stress distribution around the riveted hole has a great influence on the fatigue performance of riveted joints. The residual stresses on the contact surfaces of the plates (lower surface of the upper plate and the upper surface of the lower plate) are shown in Figure 12. It can be seen that the high-stress area of 5 mm diameter rivet is much larger than 4 mm diameter rivet.

**Figure 13:**
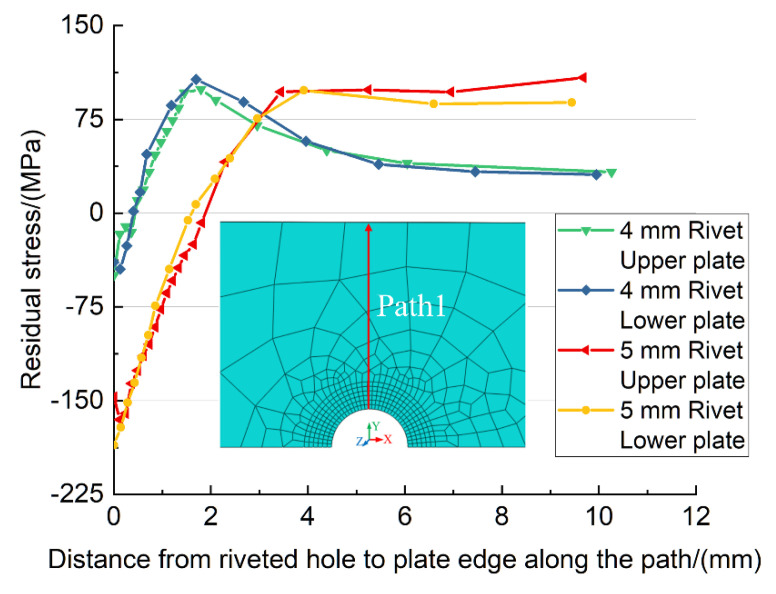
The residual stress along the path from the riveted hole to plate edge.

Furthermore, the residual stresses along the path from riveted hole to plate edge are illustrated in Figure 13. As can be seen, the residual stresses of the upper and lower plates are in good agreement at the same riveted hole. At the riveted hole area, the residual stress is compressive, then it becomes tensile as the distance from the hole wall increases. Both the maximum compressive residual stresses of 4 mm and 5 mm diameter rivets appear at the hole wall, and the value of 4 mm rivet is 3.74 times the value of 5 mm rivet. The compressive-tensile stress state transition point of the 4 mm diameter rivet occurs at 0.42 mm from the hole wall, while the transition point of the 5 mm rivet is 1.65 mm. In other words, the larger the diameter of rivet, the greater the range of compressive stress is. Moreover, the tensile residual stress of 5 mm rivet is smaller than 4 mm rivet near the riveted hole. The downward trend of 5 mm rivet is smoother than the 4 mm rivet. Although the final compressive stress of 5 mm is larger than the 4 mm rivet, it is out of the sensitive areas of stress concentration. So, it can be concluded that the use of 5 mm diameter rivet can produce a larger and broader compressive residual stress field than the 4 mm diameter rivet, which is more conducive to improving the fatigue performance of riveted lap joints.

#### 3.2.4. Analysis of Load Transfer

According to the stress distribution model in Section 2.2, it is found that changing rivet diameter and pitch will affect the transfer of rivet load and bypass load. In this section, the rivet load and bypass load of the rivets in different positions under tensile load are analyzed to verify the theoretical model.

The ratio of rivet loads of the four rivets to tensile load is shown in Table 4. The rivet loads of #1 and #4 rivets are greater than #2 and #3 rivets, which means the rivets at the two ends of the lap joint bear more load during the tensile process. This is consistent with the theoretical stress distribution analysis results. Therefore, the loading situation of 1# and 4# rivets should be of particular concern. 

The rivet loads and bypass loads of 1# and 4# rivets are exhibited in Table 5.

The group 4_4_4_4_16 is considered as a baseline group, which has the most uniformly distributed loads. The rivet loads and bypass loads between 1# and 4# rivets only differ by 6.07% and 2.09%, respectively.

To investigate the effect of rivet diameter on load transfer, group 4_4_4_5_16, group 5_4_4_4_16, and group 5_4_4_5_16 are selected to compare with the baseline group 4_4_4_4_16. For group 4_4_4_5_16, the rivet load of #1 rivet increases by 26.04%, while the #4 rivet decreases by 7.42%. The bypass load of #1 rivet decreases by 9.50%, while the #4 rivet increases by 2.5%. The variation of group 5_4_4_4_16 is similar to group 4_4_4_5_16, only the change trends of #1 and #4 rivets are opposite. For group 5_4_4_5_16, the load variations of #1 and #4 rivets are basically the same because of the same size. The increase of rivet load is more than 25%, and the reduction of bypass load is more than 8%. Therefore, a conclusion can be drawn that the increase of rivet diameter would cause a larger rivet load and smaller bypass load. In other words, the larger the rivet diameter is, the greater the load transferred by the rivet.

The effect of rivet pitch on load transfer is studied by analyzing the groups with the same rivets arrangement. The same pattern can be demonstrated from groups 4_4_4_5_x and 5_4_4_5_x that with the increase of rivet pitch, the rivet load of #1 rivet increases while the rivet load of #4 rivet decreases. The change of bypass load follows the opposite law. However, the variation law of group 5_4_4_4_x with different rivet pitches is not the same. The rivet loads of #1 rivet decrease and 4# rivet increases with the increase of rivet pitch. Moreover, the group 5_4_4_4_20 has the smallest rivet load of #1 rivet and the largest rivet load of #4 rivet. The results indicate that with the increase of rivet pitch, the rivet load gradually transferred to the rivet with a larger diameter. Moreover, if the rivets are of the same size, the rivet load would be transferred to the rivet nearest to the external tensile load (#1 rivet).

In a word, by increasing the diameter of the rivet farthest from the tensile load (#4 rivet), the stress concentration of the rivet nearest to the tensile load can be reduced, thereby the stress distribution of the riveted lap joint can be optimized. Therefore, according to the simulation analysis, the riveted joints in group 5_4_4_4_20 will have the best fatigue performance.

## 4. Experiments

The riveted lap joints specimen production and fatigue tests are carried out in this section. The theoretical model and simulation results are verified. The influence of rivet diameter and pitch on the fatigue performance of riveted joints is further investigated.

### 4.1. Experimental Procedure and Results

#### 4.1.1. Specimen Production

**Figure 14:**
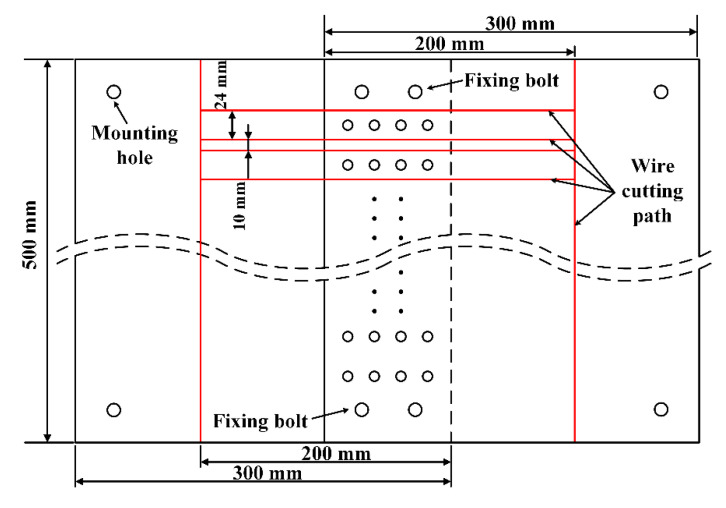
The riveted lap joint specimen to be processed.

**Figure 15:**
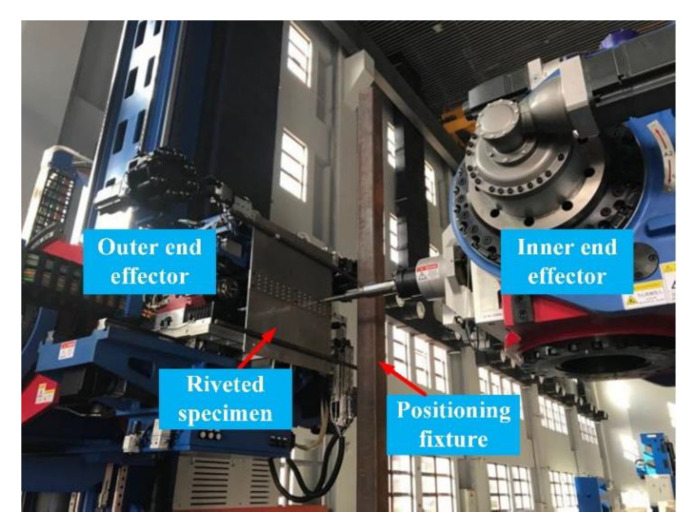
The riveted specimen production by automatic drilling and riveting system.

As shown in Figure 14, the initial riveted lap joint specimen consists of two 500 mm×300 mm×3 mm plates. The material is 2024-T3 aluminum alloy. The rivets are YSA622¨C100° countersunk rivets made of 2117-T4 aluminum alloy. The riveted joints specimen was fixed on the fixture by pre-tightening bolts and riveted by the automatic drilling and riveting machine in Figure 15. After riveting is completed, the individual samples were obtained by wire cutting.

The driven head dimensions after riveting were measured by a digital flushness gage and a vernier caliper. The measured data were averaged and the results are shown in Table 6. The experimental results of the driven head dimensions meet the riveting technical requirements. The error between simulation and experiment is within 6%. So, the finite element model can be considered correctly and effectively.

#### 4.1.2. Static Tensile Tests

**Figure 16:**
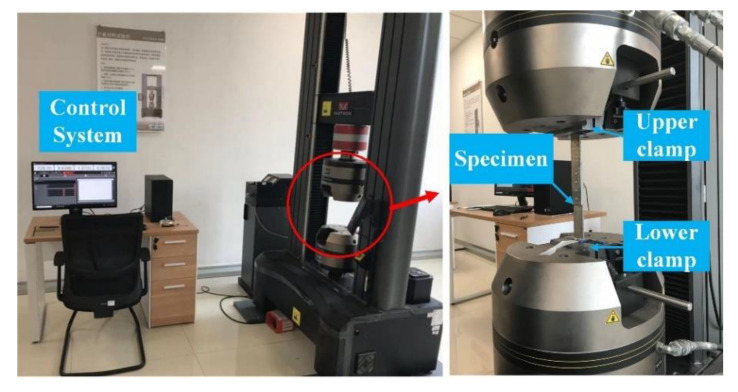
The static tensile tests using INSTRON 5985 universal testing machine.

To determine the fatigue load used in fatigue tests, the static tensile tests were carried out firs using the INSTRON 5985 universal testing machine (Instron Corporation, Boston, MA, USA). To compare the fatigue life of different groups under the same condition, the load applied to all specimens in different groups should be the same in the fatigue test. Therefore, the static tensile test is only conducted on the baseline group 4_4_4_4_16, and the obtained average tensile failure load is used for all specimens in the fatigue test. As shown in Figure 16, the riveted lap joint specimen was clamped by the fixtures. The tensile load was applied until the specimen breaks, the load recorded at the breaking moment is the static failure load $F_{b}$. The experimental results of static failure loads are listed in Table 7.

Then the fatigue load $F_{max}$ can be calculated by:

(11) $$F_{max}=0.7F_{b}.$$

#### 4.1.3. Fatigue Tests

**Figure 17:**
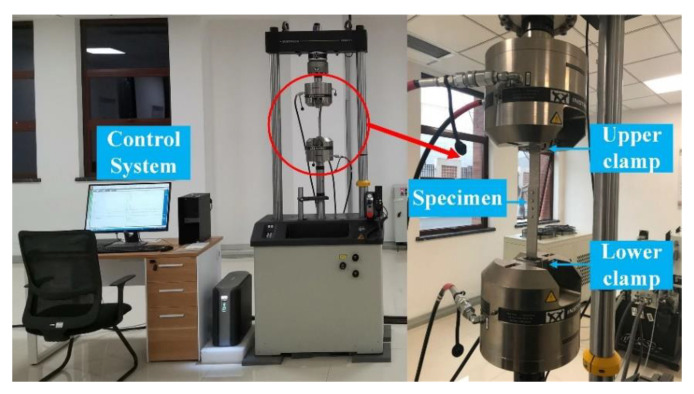
The fatigue tests performed with the INSTRON 8801 fatigue testing machine.

The fatigue tests were conducted with an INSTRON 8801 fatigue testing machine (Instron Corporation, Boston, MA, USA) as shown in Figure 17. The clamping is consistent with the tensile test. The constant amplitude load was set to $F_{max}$ which was obtained from the static tensile test. The frequency of the fatigue tests was 10 Hz and the stress ratio was set to 0.1. Each group has three specimens, so a total of 30 specimens were tested. The fatigue lives and crack sites of all riveted joints specimens are listed in Table 8.

The difference between the fatigue lives and crack sites of the specimens in the same group is mainly due to the error of the specimen production. Both the position error of rivets and the difference of interference affect the fatigue performance of the riveted specimens. 

**Figure 18:**
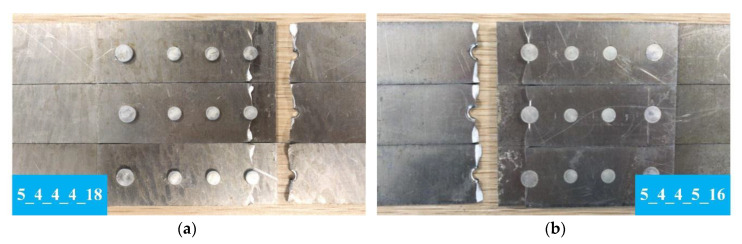
Typical fatigue crack morphology of specimens: (**a**) Group 5_4_4_4_18, crack site is on the upper plate near #1 rivet; (**b**) group 5_4_4_5_16, crack site is on the lower plate near #4 rivet.

The fractured specimens were observed as shown in Figure 18. The failure mode of all tested specimens is plate fracture. The crack sites are highly consistent, all of which occurred at the upper plate near #1 rivet and lower plate near #4 rivet. For groups 4_4_4_4_16, 4_4_4_5_x, and 5_4_4_5_x, 90.5% (19/21) of the specimens are fractured at #4 rivet area, and 88.9% (8/9) of the specimens in group 5_4_4_4_x are cracked at #1 rivet area. The results are consistent with the sensitive positions indicated by the theoretical model and finite element analysis.

### 4.2. Experimental Results Discussion

**Figure 19:**
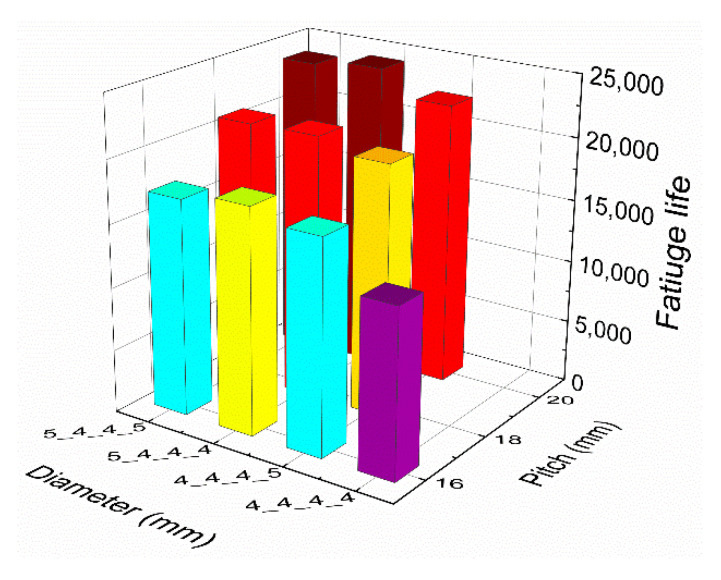
The fatigue lives of the ten groups of riveted lap joints specimens.

The average fatigue lives of the ten groups of riveted lap joints are illustrated in Figure 19. It can be demonstrated that the fatigue lives of all the other groups are improved compared to the baseline group 4_4_4_4_16. Moreover, group 5_4_4_4_20 has the longest fatigue life, which is consistent with the simulation result. Next, the effects of rivet diameter and pitch on the fatigue life of riveted lap joints are analyzed, respectively.

#### 4.2.1. Effect of Rivet Diameter on Fatigue Life of Riveted Lap Joint

**Figure 20:**
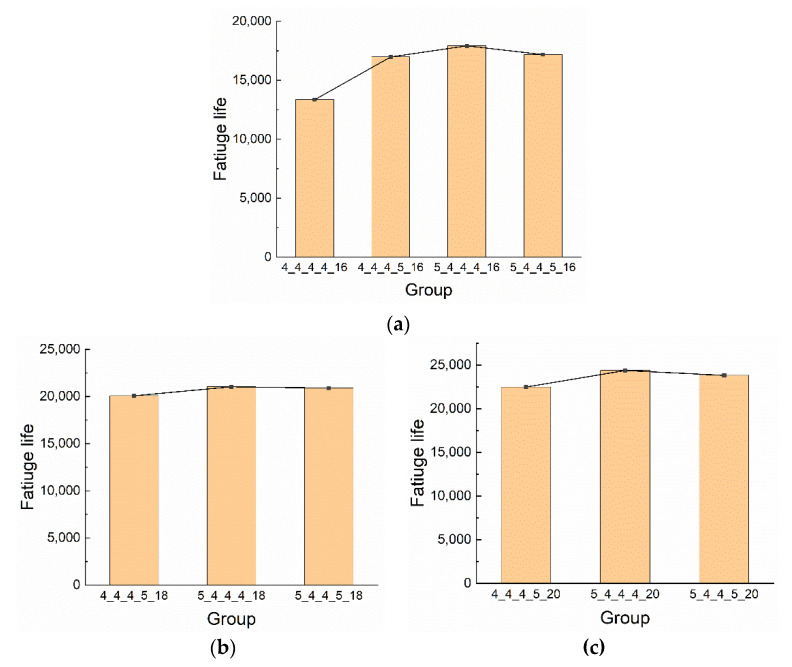
The fatigue lives of riveted lap joints with different rivet diameters: (**a**) Rivet pitch is 16 mm; (**b**) rivet pitch is 18 mm; (**c**) rivet pitch is 20 mm.

The fatigue lives of riveted lap joints with different rivet diameters are shown in Figure 20. Compared with the baseline group 4_4_4_4_16, the average fatigue lives of groups 4_4_4_5_16, 5_4_4_4_16, and 5_4_4_5_16 increased by 27.2%, 50.3%, and 68.4%, respectively. A similar increase can also be seen under the other two rivet pitches. Moreover, the fatigue life of 5_4_4_4_x group is the largest compared with groups 4_4_4_5_x and 5_4_4_5_x, which is consistent with the simulation results. 

#### 4.2.2. Effect of Rivet Pitch on Fatigue Life of Riveted Lap Joint

**Figure 21:**
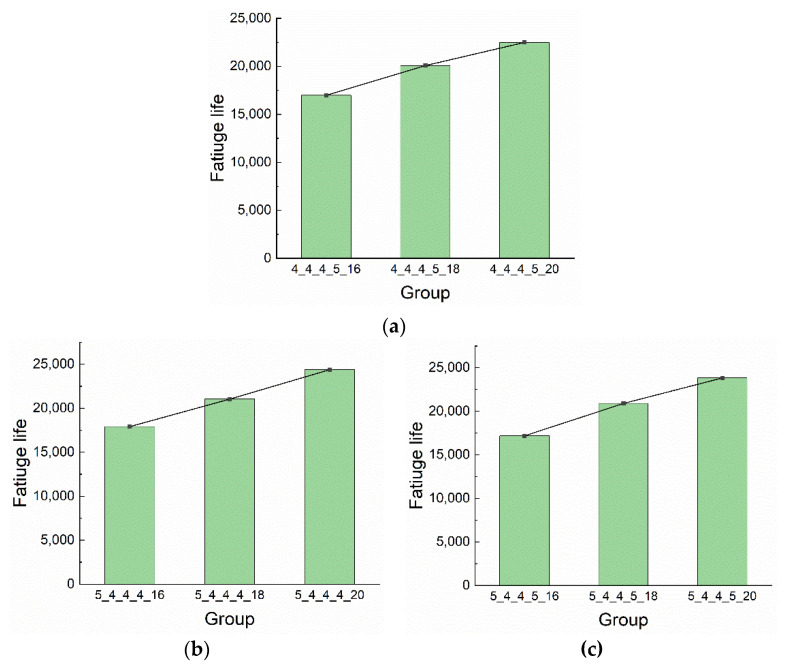
The fatigue lives of riveted lap joints with different rivet pitches: (**a**) groups 4_4_4_5_x; (**b**) groups 5_4_4_4_x; (**c**) groups 5_4_4_5_x.

Figure 21 exhibits the fatigue lives of riveted lap joints with different rivet pitches. In groups 4_4_4_5_x, the fatigue lives of 18 mm and 20 mm rivet pitch groups increased by 18.16% and 32.28% compared with the 16 mm rivet pitch group, respectively. In groups 5_4_4_4_x, the increase of 18 mm and 20 mm groups are 17.41% and 36.10% compared with the 16 mm group, respectively. Similarly, the fatigue lives of 18 mm and 20 mm rivet pitches in groups 5_4_4_5_x are increased by 21.61% and 38.62% than the 16 mm group, respectively. In general, with the increase of rivet pitch, the fatigue life of the riveted specimen increases. Therefore, the riveted lap joint has the best fatigue performance when the rivet pitch is 20 mm rather than 16 mm or 18 mm.

In summary, the experimental results verified the correctness of the theoretical analysis and simulation results. The group 5_4_4_4_20 has the longest fatigue life and can be considered as the optimal structural parameters of riveted lap joints. 

## 5. Conclusions

In this paper, the stress distribution model of the riveted lap joint concerning rivet load and bypass load is established. The combined effect of the rivet diameters and pitches on the residual stress and load transfer of the riveted lap joint is numerically studied. The fatigue tests are carried out to investigate the fatigue life of riveted specimens with various parameters. The experimental results agree well with theoretical analysis and simulation results. The conclusions of this paper are drawn as follows:

1. The difference of bypass load at different positions is an important factor affecting the stress distribution of the riveted lap joint. Moreover, the rivet load and bypass load can be redistributed by optimizing the rivet diameters and pitches, which is beneficial to the fatigue performance of riveted joints.
2. The rivet with a larger diameter can produce a larger and broader compressive residual stress field. By increasing the diameter of the rivet farthest from the tensile load, the stress concentration of the rivet nearest to the tensile load can be reduced, thereby the stress distribution of the riveted lap joint can be optimized.
3. With the increase of rivet pitch, the rivet load gradually transferred to the rivet with a larger diameter. Moreover, if the rivets are of the same size, the rivet load will transfer to the rivet nearest to the external tensile load.
4. In all ten groups of riveted lap joints with different rivet diameters and pitches, the group 5_4_4_4_20 has the longest fatigue life and can be considered as the optimal structural design of riveted lap joints.

## Figures and Tables

**Table 1:** The ten groups of riveted lap joints with different rivet diameters and pitches (unit: mm).

Group	Rivet Diameter of #4	Rivet Diameter of #3	Rivet Diameter of #2	Rivet Diameter of #1	Rivet Pitch
4_4_4_4_16	4	4	4	4	16
5_4_4_4_16	5	4	4	4	16
5_4_4_4_18	5	4	4	4	18
5_4_4_4_20	5	4	4	4	20
4_4_4_5_16	4	4	4	5	16
4_4_4_5_18	4	4	4	5	18
4_4_4_5_20	4	4	4	5	20
5_4_4_5_16	5	4	4	5	16
5_4_4_5_18	5	4	4	5	18

**Table 2:** Material properties for 2024-T3 plates and 2117-T4 rivets [20].

Material Parameter	2024-T3	2117-T4
Young’s modulus, *E*	72.4 GPa	71.7 GPa
Poisson’s ratio, *v*	0.33	0.33
Yield stress, $\sigma_{s}$	310 MPa	172 MPa
Strength coefficient, *C* ^1^	745 MPa	544 MPa
Hardening exponent, *m* ^1^	0.164	0.15

^1^ The stress–strain relationship is described by Hollomon constitutive model $\sigma =C\cdot {\left(\varepsilon \right)}^{m}$.

**Table 3:** The driven head dimensions obtained from simulation results (unit: mm).

	Diameter of Driven Head		Height of Driven Head	
	Standard ^1^	Simulation	Standard ^1^	Simulation
4	6.0 ± 0.4	6.29	≥1.6	2.75
5	7.5 ± 0.5	7.71	≥2.0	3.52

^1^ The standard values of the driven head dimensions refer to [19].

**Table 4:** The ratio of rivet loads to tensile load of different groups of riveted lap joints.

Group	#4 Rivet	#3 Rivet	#2 Rivet	#1 Rivet
4_4_4_4_16	25.20%	24.51%	23.56%	26.73%
5_4_4_4_16	23.33%	21.90%	21.08%	33.69%
5_4_4_4_18	22.15%	21.39%	20.86%	35.61%
5_4_4_4_20	20.66%	21.63%	20.94%	36.77%
4_4_4_5_16	31.33%	21.84%	22.00%	24.83%
4_4_4_5_18	33.68%	21.75%	21.71%	22.86%
4_4_4_5_20	34.23%	21.89%	21.93%	21.96%
5_4_4_5_16	31.55%	16.42%	18.10%	33.93%
5_4_4_5_18	30.27%	16.58%	17.57%	35.57%
4_4_4_4_16	28.59%	17.08%	18.01%	36.32%

**Table 5:** The rivet loads and bypass loads of 1# and 4# rivets (unit: N).

Group	Rivet Load of #4 Rivet	Variation ^1^	Rivet Load of #1 Rivet	Variation ^1^	Bypass Load of #4 Rivet	Variation ^1^	Bypass Load of #1 Rivet	Variation ^1^
4_4_4_4_16	2671.20	-	2833.38	-	7928.80	-	7766.62	-
4_4_4_5_16	2472.98	−7.42%	3571.14	26.04%	8127.02	2.50%	7028.86	−9.50%
4_4_4_5_18	2347.60	−12.11%	3774.18	33.20%	8252.40	4.08%	6825.82	−12.11%
4_4_4_5_20	2189.43	−18.04%	3898.04	37.58%	8410.57	6.08%	6701.96	−13.71%
5_4_4_4_16	3320.98	24.33%	2631.98	−7.11%	7279.02	−8.20%	7968.02	2.59%
5_4_4_4_18	3570.50	33.67%	2423.24	−14.48%	7029.50	−11.34%	8176.76	5.28%
5_4_4_4_20	3628.10	35.82%	2327.26	−17.86%	6971.90	−12.07%	8272.74	6.52%
5_4_4_5_16	3344.57	25.21%	3596.10	26.92%	7255.44	−8.49%	7003.90	−9.82%
5_4_4_5_18	3208.80	20.13%	3770.84	33.09%	7391.20	−6.78%	6829.16	−12.07%
5_4_4_5_20	3030.18	13.44%	3850.09	35.88%	7569.82	−4.53%	6749.91	−13.09%

^1^ The variation is compared to the baseline group 4_4_4_4_16.

**Table 6:** The driven head dimensions obtained from experimental results (unit: mm).

Rivet Diameter	Diameter of Driven Head			Height of Driven Head		
	Simulation	Experiment	Error	Simulation	Experiment	Error
4	6.29	6.21	1.27%	2.75	2.87	4.36%
5	7.71	7.56	1.95%	3.52	3.71	5.40%

**Table 7:** The tests result of static tensile failure load *F_b_* (kN).

Group	Specimen 1	Specimen 2	Specimen 3	Average
4_4_4_4_16	15.250	15.147	15.188	15.195

**Table 8:** The fatigue lives and crack sites of the riveted lap joints.

Group	Specimen	Fatigue Life	Average	Crack Site
4_4_4_4_16	1	12,867	13,355	lower plate near #4 rivet
	2	14,449		upper plate near #1 rivet
	3	12,748		lower plate near #4 rivet
4_4_4_5_16	1	16,047	16,986	lower plate near #4 rivet
	2	16,045		lower plate near #4 rivet
	3	18,867		lower plate near #4 rivet
4_4_4_5_18	1	18,176	20,070	lower plate near #4 rivet
	2	21,874		lower plate near #4 rivet
	3	20,161		lower plate near #4 rivet
4_4_4_5_20	1	23,966	22,487	lower plate near #4 rivet
	2	23,260		lower plate near #4 rivet
	3	20,236		upper plate near #1 rivet
5_4_4_4_16	1	17,801	17,920	upper plate near #1 rivet
	2	17,202		upper plate near #1 rivet
	3	18,756		upper plate near #1 rivet
5_4_4_4_18	1	22,082	21,037	upper plate near #1 rivet
	2	18,252		upper plate near #1 rivet
	3	22,777		upper plate near #1 rivet
5_4_4_4_20	1	25,171	24,389	upper plate near #1 rivet
	2	21,421		upper plate near #1 rivet
	3	26,574		lower plate near #4 rivet
5_4_4_5_16	1	19,961	17,183	lower plate near #4 rivet
	2	15,148		lower plate near #4 rivet
	3	16,441		lower plate near #4 rivet
5_4_4_5_18	1	20,986	20,897	lower plate near #4 rivet
	2	20,580		lower plate near #4 rivet
	3	21,126		lower plate near #4 rivet
5_4_4_5_20	1	26,751	23,819	lower plate near #4 rivet
	2	24,121		lower plate near #4 rivet
	3	20,585		lower plate near #4 rivet
